# Distribution and fibrotic response following inhalation exposure to multi-walled carbon nanotubes

**DOI:** 10.1186/1743-8977-10-33

**Published:** 2013-07-30

**Authors:** Robert R Mercer, James F Scabilloni, Ann F Hubbs, Lori A Battelli, Walter McKinney, Sherri Friend, Michael G Wolfarth, Michael Andrew, Vincent Castranova, Dale W Porter

**Affiliations:** 1 Health Effects Laboratory Division, NIOSH, MS 2015, 1095 Willowdale Drive, Morgantown, WV, USA; 2 Department of Physiology and Pharmacology, West Virginia University, Morgantown, WV, USA

## Abstract

**Background:**

Prior studies have demonstrated a rapid and progressive acute phase response to bolus aspiration of multi-walled carbon nanotubes (MWCNTs). In this study we sought to test the hypothesis that inhalation exposure to MWCNT produces a fibrotic response and that the response is chronically persistent. To address the hypothesis that inhaled MWCNTs cause persistent morphologic changes, male C57BL/6 J mice were exposed in a whole-body inhalation system to a MWCNT aerosol and the fibrotic response in the alveolar region examined at up to 336 days after termination of exposure.

**Methods:**

Inhalation exposure was to a 5 mg/m^3^ MWCNT aerosol for 5 hours/day for 12 days (4 times/week for 3 weeks). At the end of inhalation exposures, lungs were either lavaged for analysis of bronchoalveolar lavage (BAL) or preserved by vascular perfusion of fixative while inflated with air at 1, 14, 84, 168 and 336 days post inhalation exposure. Separate, clean-air control groups were also studied. Light microscopy, enhanced darkfield microscopy and field emission electron microscopy (FESEM) of tissue sections were used to analyze the distribution of lung burden following inhalation exposure. Morphometric measurements of Sirius Red staining for fibrillar collagen were used to assess the connective tissue response. Serial section analysis of enhanced darkfield microscope images was used to examine the redistribution of MWCNT fibers within the lungs during the post-exposure period.

**Results:**

At day 1 post-exposure 84 ± 3 and 16 ± 2 percent of the lung burden (Mean ± S.E., N = 5) were in the alveolar and airway regions, respectively. Initial distribution within the alveolar region was 56 ± 5, 7 ± 4 and 20 ± 3 percent of lung burden in alveolar macrophages, alveolar airspaces and alveolar tissue, respectively. Clearance reduced the alveolar macrophage burden of MWCNTs by 35 percent between 1 and 168 days post-exposure, while the content of MWCNTs in the alveolar tissue increased by 63 percent. Large MWCNT structures containing greater than 4 fibers were 53.6 percent of the initial lung burden and accounted for the majority of the decline with clearance, while lung burden of singlet MWCNT was essentially unchanged. The mean linear intercept of alveolar airspace, a measure of the expansion of the lungs, was not significantly different between groups. Pulmonary inflammation and damage, measured as the number of polymorphnuclear leukocytes (PMNs) or lactate dehydrogenase activity (LDH) and albumin in BAL, increased rapidly (1 day post) after inhalation of MWCNTs and declined slowly with time post-exposure. The fibrillar collagen in the alveolar region of MWCNT-exposed mice demonstrated a progressive increase in thickness over time (0.17 ± 0.02, 0.22 ± 0.02, 0.26 ± 0.03, 0.25 ± 0.02 and 0.29 ± 0.01 microns for 1, 14, 84, 168 and 336 days post-exposure) and was significantly different from clean-air controls (0.16 ± 0.02) at 84 and (0.15 ± 0.02) at 336 days post-exposure.

**Conclusions:**

Despite the relatively low fraction of the lung burden being delivered to the alveolar tissue, the average thickness of connective tissue in the alveolar region increased by 70% in the 336 days after inhalation exposure. These results demonstrate that inhaled MWCNTs deposit and are retained within the alveolar tissue where they produce a progressive and persistent fibrotic response up to 336 days post-exposure.

## Background

A number of studies have used inhalation exposure of experimental animals to assess the health risks of multi-walled carbon nanotubes (MWCNTs). Acute inhalation studies have generally studied the pulmonary and systemic responses without a significant recovery in the post-exposure period following exposure. For instance, Li *et al.*[1] found no significant inflammation and thickening of the alveolar septa in studies at 32 mg/m^3^ for periods varying from 8 to 24 days with sacrifice at the end of exposure. Mitchell *et al.*[2] did a range of inhalation exposures from 0.3 to 5 mg/m^3^ for 7 and 14 days with sacrifice the day after exposure termination. While nonmonotonic systemic immunosuppression after 14 days was observed, no significant histopathology was observed in these acute exposures. In contrast, an acute mouse inhalation study conducted in our laboratory at 10 mg/m^3^ for 2, 4, 8 or 12 days demonstrated dose-dependent pulmonary inflammation and the rapid development of a fibrotic response [3].

Ryan-Rasmussen *et al.*[4] conducted MWCNT aerosol exposures in control and ovalbumin-sensitized mice at 100 mg/m^3^ for 6 hours with recovery periods of 1 and 14 days after inhalation. Fibrotic responses were found in the tissues surrounding airways of ovalbumin-sensitized mice, and there was elevation in whole lung soluble collagen but no significant changes were observed in unsensitized mice exposed to MWCNT aerosol. Rats exposed to MWCNT aerosols up to 2.5 mg/m^3^ for 13 weeks showed multifocal granulomatous inflammation, diffuse histiocytic and neutrophilic inflammation, and intra-alveolar lipoproteinosis [5], while another 13 week inhalation study of rats to MWCNT reported persistent pulmonary inflammation and granulomas in rats exposed to 0.4 – 6 mg/m^3^[6].

We have previously reported the distribution and fibrotic response following aspiration of MWCNTs with analysis of the post-exposure response up to 56 days in mice [7,8]. In that study, there was an initial inflammatory phase observed in the lungs which lasted approximately 2 weeks. Administration of the MWCNTs by aspiration was conducted with the aid of a previously described dispersion solution [9], which mimicked the contents of the lungs liquid lining layer and resulted in absence of large agglomerates of MWCNTs in the lungs following aspiration. As a result, granulomatous lesions were only rarely observed, and there were no large, airspace agglomerates of MWCNTs in the lungs. The majority of the initial MWCNT lung burden was demonstrated to be in alveolar macrophages. The alveolar interstitium received approximately 8% of the MWCNT lung burden, which was generally in the form of singlets or small, micron diameter MWCNT structures containing only a few fibers. There was development of a fibrotic response in the alveolar interstitium that progressed throughout the 56 day post-exposure period and was observed to be directly correlated to the presence of MWCNTs in the alveolar interstitium.

In this study, we sought to compare the responses between aspiration and inhalation and to extend the analysis out to a post-exposure response of 336 days using a recently developed inhalation exposure system for whole body exposure [10]. This system is capable of delivering a well dispersed aerosol to optimize the alveolar level lung burden without deposition of large, airspace masses.

The use of a well dispersed aerosol establishes a uniform initial distribution of MWCNTs within the lungs. The number of fibers per MWCNT structure was measured in the lungs to address the issue of redistribution of MWCNTs during the ensuing post-exposure period. The number distribution of fibers/nanotubes per MWCNT structure at different times during the post-exposure period was determined by using a combination of morphometry, serial sections and enhanced darkfield microscopy to count the number of MWCNT structures in each size class. Although limited to classifying MWCNT structures containing one, two, three, four and greater than four fibers per structure, our prior studies [11,12] indicate that the smaller MWCNT structures are the ones which are found within the alveolar interstitium and may play a critical role in the fibrotic response.

## Results

**Figure 1:**
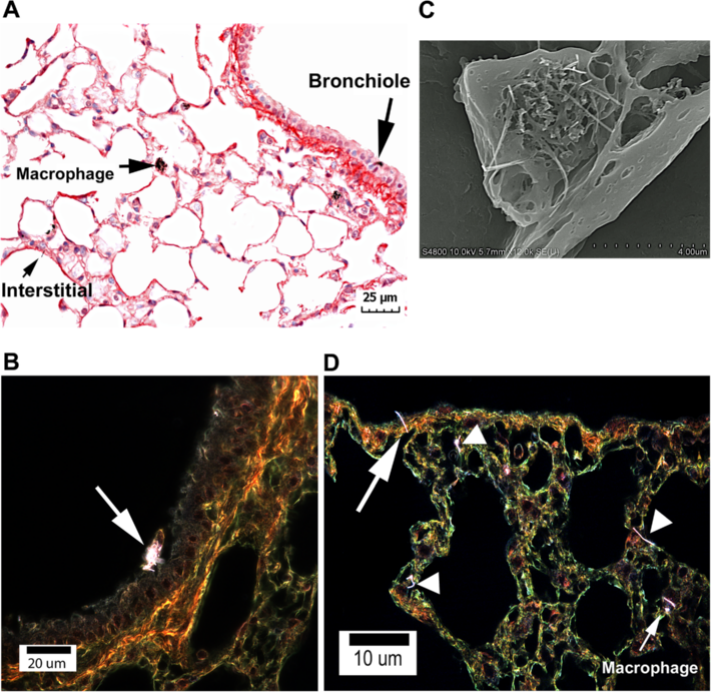
**Micrographs of illustrating distribution of MWCNTs in the lungs 1 day after termination of inhalation exposure.** Examples of MWCNTs (arrows) in the three highest initial sites of deposition, alveolar macrophage, alveolar interstitium and bronchiole, are illustrated by the light micrograph of **A**. A MWCNT (white fibers)-loaded macrophage is shown in the enhanced darkfield image of **B**. The FESEM of **C** shows a section through a MWCNT-loaded alveolar macrophage. The enhanced darkfield image of **D** shows a MWCNT penetration of the visceral pleura (arrow) and other MWCNTs in the alveolar interstitium (tailless arrows). MWCNT in an alveolar macrophage indicated by smaller arrow.

Figure 1 shows micrographs from various imaging methods, which demonstrate the distribution of MWCNTs in the lungs 1 day after the termination of the 12 day inhalation exposure period. Figure 1A is a light micrograph of a Sirius Red stained section showing MWCNTs in a bronchiole, alveolar macrophage, and a case of a fiber midway in penetration between the alveolar airspace and alveolar epithelial surface. Granulomatous responses surrounding airspace MWCNT structures were not observed after inhalation of the well dispersed MWCNT aerosol. Figure 1B shows a MWCNT-loaded macrophage in a bronchiole of the lungs. An FESEM image of a MWCNT-loaded alveolar macrophage is shown in Figure 1C. No large, airspace clumps of MWCNTs or granulomatous lesions surrounding MWCNT masses were observed. Penetrations of the visceral pleura by MWCNT fibers (Figure 1D, arrow) were also observed in inhalation-exposed lungs similar to that previously reported for aspiration exposure [13] and acute inhalation exposure [3].

**Figure 2:**
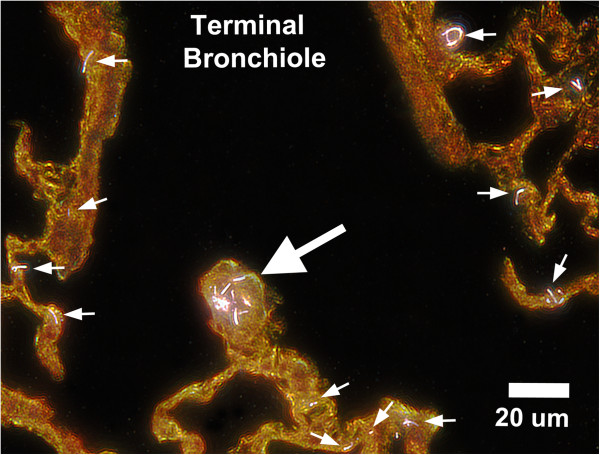
**Enhanced darkfield image of the transition region between terminal bronchiole and first alveolar duct bifurcation at 168 days after exposure to MWCNT.** The large arrow in this micrograph indicates a cluster of MWCNTs (white fibers) in the ridge of the first alveolar duct bifurcation. Smaller arrows indicate some of the numerous singlet and small MWCNT structures distributed throughout the alveolar septa of this critical transition region between conducting airways and gas exchange regions of the lungs.

At 14 days and later times post-exposure, MWCNT-loaded alveolar macrophages, similar to Figure 1C and singlet or small clusters of MWCNTs in the alveolar tissue were the most prominent compartments in which MWCNTs were observed. The enhanced darkfield image of Figure 2 shows singlet MWCNTs and small clusters of MWCNTs within alveolar septa of the transition region between a terminal bronchiole and first alveolar duct bifurcation at 168 days after exposure to MWCNTs. As was typically observed at 14 days and later times, a cluster of MWCNTs are present within the ridge of the first alveolar duct bifurcation of Figure 2. Inhaled particles on the ridge of the first alveolar duct bifurcation are the principal site of deposition following inhalation exposures to particles [14] and asbestos fibers [15]. Typically in these inhalation studies, particles or fibers are densely focused on the ridge of the first alveolar duct bifurcation, are proportionately lower in subsequent alveolar duct bifurcations, and are only sparsely found in alveolar septa adjacent to alveolar duct bifurcation ridges. As shown in Figure 2, inhaled MWCNTs also produced a high concentration of fibers on the bifurcation ridge. In addition, singlet or small MWCNT structures were consistently observed in adjacent alveolar septa (small arrows) and throughout the more distal alveolar septa of the lungs. The occurrence of MWCNTs throughout alveolar tissues of the lungs is unlike that observed for other particles.

**Figure 3:**
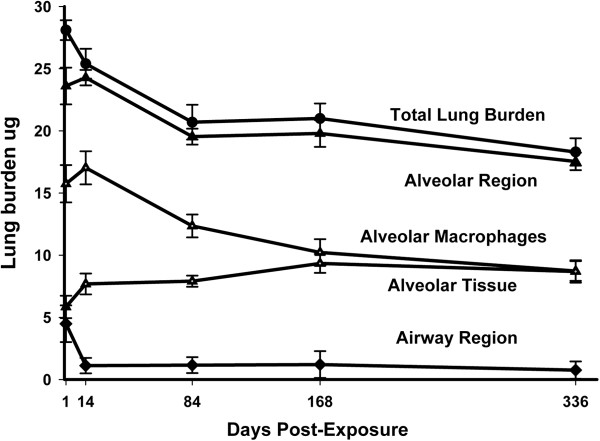
**Changes in the distribution of MWCNTs in the lungs after inhalation exposure to MWCNTs.** Changes in the lung burden are given for total lung burden, the airway region and the alveolar region. Burden in the alveolar region is further subdivided into that present in alveolar macrophages and that in alveolar tissue (including MWCNTs in subpleural alveolar tissue). Between day 1 and 14 days post-exposure the burden in the airway region rapidly declined to a near steady level for the remainder of the post-exposure period. The burden in the alveolar region paralleled the total lung burden with the small difference being due to that present in the airway region. Burden in alveolar macrophages was initially 3 fold higher than burden in the alveolar tissue but was nearly equal by 336 days post-exposure as the burden in alveolar macrophages declined and the burden in alveolar tissue increased. Data Mean ± S.E., N = 5.

The lung burden of MWCNTs in airway and alveolar region components of the lungs at 1, 14, 84, 168 and 336 days post-exposure are given in Figure 3. Initially, MWCNTs in the airways accounted for 16 percent of the initial distribution with 84 percent in the alveolar region (including 1.2 percent of MWCNTs in the subpleural tissue region). At 336 days, 4.2 percent of the initial lung burden was found in the airways and 95.8 percent of the initial lung burden remained in the alveolar region (including 4.8 percent in the subpleural tissue region). Alveolar macrophages initially contained nearly 3 fold the burden present in the alveolar tissue and also had the greatest clearance, declining from 15.8 ug at 1 day post-exposure to 10.2 ug at 168 days post-exposure. Over the same period, the content of MWCNTs in the alveolar tissue increased from 5.8 ug at 1 day post-exposure to 9.5 ug at 168 days post-exposure. The airway region, after an initial rapid decline between 1 and 14 days post-exposure, contained a nearly constant, low level of burden between day 14 and 168 days post-exposure. Total lung burdens of MWCNTs were 28.1 ± 0.8, 25.4 ± 1.2, 20.7 ± 1.4, 21.0 ± 1.2, and 18.3 ± 1.1 ug (Mean ± S.E., N = 8) at 1, 14, 84, 168 and 336 days, respectively (Mean ± S.E., N = 8). A highly significant (P < 0.0001) decrease in MWCNT lung burden with post-exposure time was determined.

**Figure 4:**
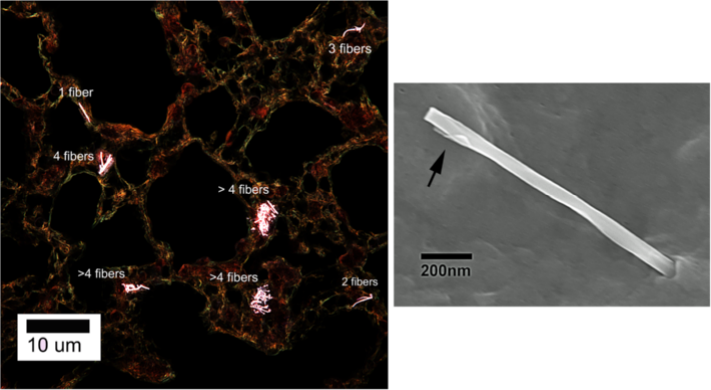
**Examples of varying fiber numbers for MWCNT structures.** Left, enhanced darkfield image (1 day post-exposure) shows examples of MWCNT structures containing 1, 2, 3, 4 and >4 fibers per structure. MWCNT fibers (and most nanoparticles in general) are bright white in enhanced darkfield images due to scattering of light while tissue is dark and airspaces are black. FESEM micrograph on right shows an example of a singlet MWCNT penetration of the alveolar epithelium. Arrow in the FESEM indicates the end of the singlet that is in the airspace where partial shells of the MWCNT are visible. FESEM is from 168 days post-exposure.

Examples of MWCNT structures containing 1, 2, 3, 4 and >4 fibers per MWCNT structure are shown in the enhanced darkfield image of Figure 4 (upper image) taken from a 1 day post-exposure lung. Counting of these cases was based on using serial sections to avoid bias of selecting larger fibers and to accurately count the number of fibers per structure and thus determine how the distribution of MWCNT fibers changed once deposited in the lungs. MWCNT fibers and most nanoparticles in general, are efficient at scattering light over a broad wavelength. When the scattered light is imaged by an enhanced darkfield microscope, the fibers appear bright white due to scattering of light, while tissue is dark and airspaces are black. This contrast significantly enhances the ability to detect isolated or rare nanoparticles. The right hand, FESEM image of Figure 4 is an example of a singlet MWCNT penetration of the alveolar epithelium at 168 days post-exposure. Partial shells of the MWCNT structure are visible at the airspace end (arrow).

**Figure 5:**
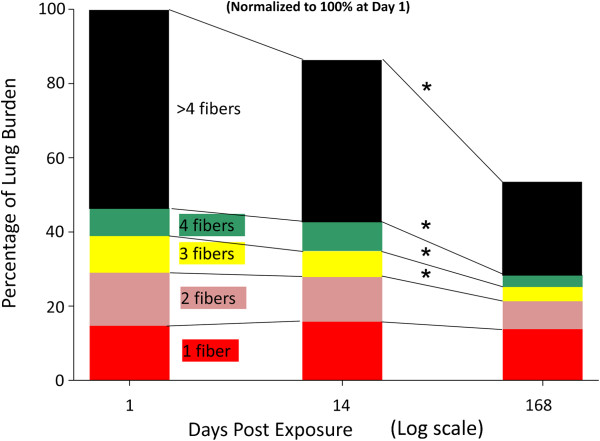
**Changes in the distribution of MWCNT lung burden between 1 day and 168 days post-exposure.** The majority of MWCNT structures initially in the lungs contained 5 or more fibers and were reduced from 53.6 ± 2.3 at 1 day post-exposure to 25.3 ± 2.8 at 168 days (Mean ± S.E., N = 7). Significant reductions were also found in MWCNT structures containing 2, 3 or 4 fibers. The lung burden of singlets (1 fiber) as a percentage of the initial total lung burden did not significantly change from 1 day to 168 days post-exposure. Asterisks significantly different from day 1, p < 0.05.

Measurements of the fiber number per MWCNT structure at 1, 14 and 168 days after the termination of inhalation exposure are given in Figure 5. The results given in Figure 5 demonstrate that clearance depends on the number of fibers in the MWCNT structure. Large agglomerates (>4 fibers/ MWCNT structure) account for the majority of MWCNT clearance from the lungs. From day 1 to 168 days, 28.3 percent of the initial lung burden was cleared from the lungs by removal of large agglomerate (>4 fibers/MWCNT structure). The initial lung burden present as singlet MWCNTs was essentially unchanged over the 168 day post-exposure period. Intermediate percentages of initial lung burden decreasing in the lungs in the cases of 2, 3 and 4 fibers per MWCNT structure. Total MWCNT fiber number of the initial lung burden was 1,321 million based on an initial lung burden of 28.1 ug/lung and a conversion of 47 million MWCNT fibers per ug [16].

**Figure 6:**
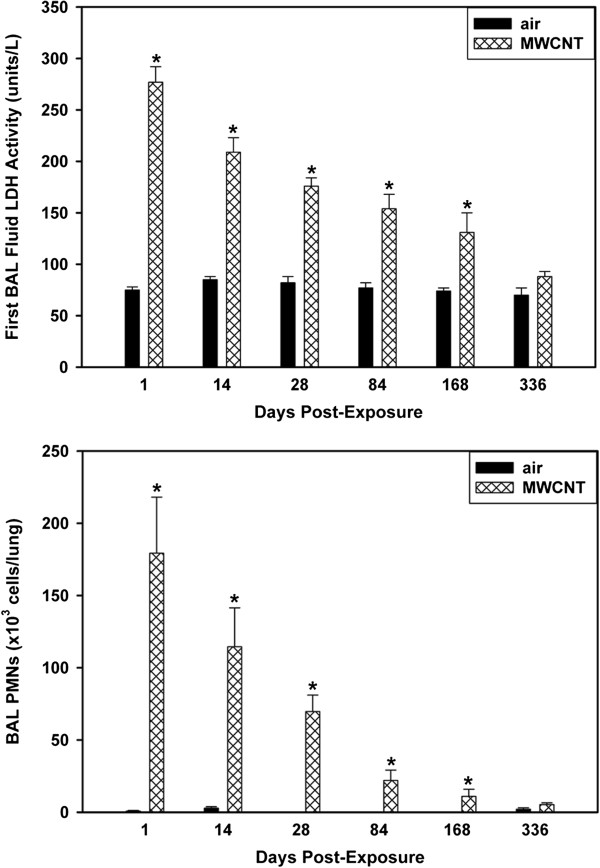
**BAL analysis of pulmonary injury and inflammation.** Mice were exposed by inhalation exposure to air (vehicle) or MWCNTs (5 mg/m^3^) for 12 days. BAL LDH and BAL PMNs studies were conducted at 1, 14, 28, 84, 168 and 336 days post-exposure. Cytotoxicity was assessed by BAL fluid LDH activities and pulmonary inflammation by BAL polymorphonuclear leukocytes. BAL fluid LDH activities (upper panel) and BAL PMNs (lower panel) were significantly elevated above respective clean-air controls from 1 to 168 days post-exposure (Mean ± S.E., N = 6-8). An asterisk indicates air-exposed controls were significantly lower (p < 0.05) in comparison to the corresponding MWCNT-exposed group. Highly significant decreases in LDH (P < 0.0001) and BAL PMNs (P < 0.002) with post-exposure time were determined. BAL protein was also measured and followed the same pattern as LDH (data not shown).

MWCNT-induced cytotoxicity in the lung was assessed by determining BAL fluid LDH activities (Figure 6A) and albumin while pulmonary inflammation was assessed by determining BAL polymorphonuclear leukocytes (PMNs) (Figure 6B). The time course of albumin in first BAL fluid followed the same pattern as that of LDH activity (data not shown). MWCNT-exposed mice had significantly higher BAL fluid LDH activities, albumin and BAL PMN levels in comparison to the corresponding air-exposed mice at all post-exposure times. For mice exposed to MWCNTs, BAL fluid LDH activities and BAL PMNs decreased significantly with post-exposure time, but statistically significantly different from controls out to 168 days. At 336 days post-exposure BAL fluid LDH activities, albumin and BAL PMN levels still elevated above controls but no longer statistically different.

**Figure 7:**
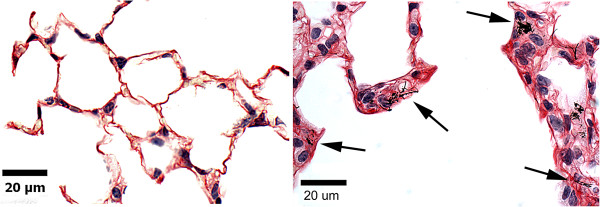
**Light microscopy images of collagen fibers in clean-air control and MWCNT-exposed lungs.** Left micrograph shows normal clean-air control section of the alveolar region. Right micrograph is from fibrotic region of a MWCNT-exposed lung at 168 days post-exposure. Arrows indicate some of the MWCNT structures within the alveolar interstitium in this region. Fibrillar collagen, stained with Sirius Red. Nuclei counterstained with Hematoxylin.

Figure 7 shows representative light micrographs of Sirius Red stained sections from a clean-air control lung and a lung at 168 days after MWCNT inhalation. As illustrated in Figure 7, areas of the lung where MWCNT fibers were observed in the interstitial space were found to develop dense bands of fibrillar collagen (arrows). In our prior study using aspiration exposure to MWCNTs, sites with concentrations of interstitial MWCNTs were also found to be foci for a fibrotic response [7,8].

**Figure 8:**
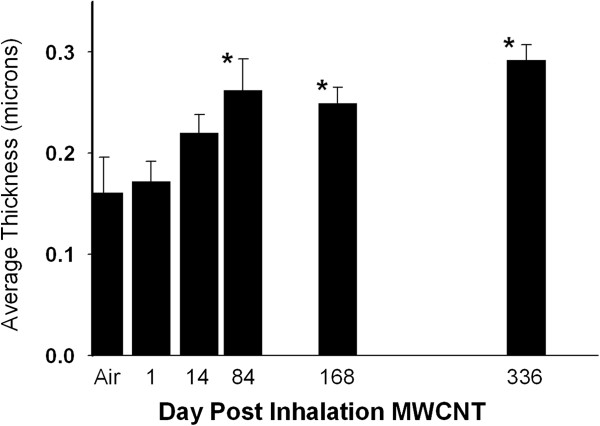
**Morphometric determination of fibrillar collagen thickness response to inhaled MWCNT exposure.** The average thickness of fibrillar collagen in the alveolar septa was increased by approximately 70 percent by 336 days post-exposure. Asterisks significantly different from day 1, p < 0.05. (Mean ± S.E., N = 6).

Morphometric assessment of the fibrillar collagen response, based on Sirius Red binding to fibrillar collagen, at 1, 14, 84, 168 and 336 day post-exposure is given in Figure 8. The average thickness of fibrillar collagen for the clean-air controls was 0.16 ± 0.02 um. In a prior morphometric study, where high resolution transmission electron microscopy imaging was used to identify and quantify fibrillar collagen, at a resolution of individual fibrils, a value of 0.15 ± 0.02 um was obtained in comparable body weight of CD-1 mice [17]. Morphometric measurements of that study were shown to be comparable, albeit approximately 30 percent lower, than to those obtained by biochemical measurements. Biochemical measurement of whole lung or lobe fibrillar collagen also include the connective tissue (fibrillar collagen and elastin) of the airway, blood vessels and their associated bronchovascular cuff. Biochemical measurements significantly over-estimate collagen in the alveolar region and are relatively insensitive to changes in the alveolar region as these non-alveolar areas are rich in fibrillar collagen and contain 68% of the total tissue mass of the lungs and approximately 70% of total lung connective tissue [18].

A trend toward increased fibrillar collagen within alveolar septa is apparent at 14 days post-exposure, which is consistent with our earlier observations of a rapid fibrotic response in acute inhalation exposure to MWCNTs [3]. The increase in fibrillar collagen was significantly elevated over control at 84, 168 and 336 days post-exposure and increases by 62, 56 and 70% above 1 day post-exposure, respectively. Despite the increased thickness of the alveolar fibrillar collagen, mean linear intercept length, a measure of the average size of airspaces in the alveolar region of the lungs was 29.5 ± 0.5, 29.6 ± 0.6, 29.1 ± 0.5, 29.7 ± 0.4, 29.5 ± .0.2 and 29.1 ± .0.2 and 29.5 ± .2 microns for clean-air control, 1 day, 14 day, 84 day and 168 day post-exposure MWCNT-exposed groups. Mean linear intercept length were not significantly different between groups.

**Figure 9:**
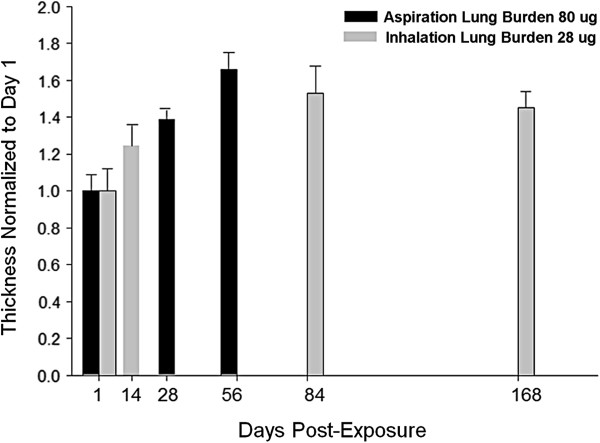
**Comparison of fibrillar collagen response to inhaled versus bolus aspiration of MWCNTs.** Although the total lung burden of these two routes differs by a factor of approximately 3, the time course and magnitude of response to bolus aspiration and inhalation exposure of MWCNT to the lungs are remarkable similar. Therefore, the fibrogenic potency of inhaled MWCNTs is approximately 3-fold greater than aspirated MWCNT (bolus exposure) on an equivalent lung burden basis. Fibrillar collagen response data following bolus aspiration exposure to MWCNTs from Mercer *et al*. [7], Figure 6 (Mean ± S.E., N = 6).

A comparison of the fibrillar collagen response between our earlier study [11] using aspiration delivery of MWCNTs to the present inhalation exposure is given in Figure 9. The earlier study, Figure 6 in Mercer *et al*. [11], used the same morphometric methods to determine the alveolar interstitial collagen fibers response to MWCNT, expressed as the average thickness of connective tissue in the alveolar interstitium, from 1 to 56 days after bolus aspiration.

The time course of response of bolus aspiration and inhalation exposure of MWCNTs to the lungs is similar in spite of the differences in the rate of exposure, bolus versus 12 day exposure period. The magnitudes of alveolar interstitial fibrosis for aspiration vs. inhalation of MWCNTs appear similar in Figure 9. However, it should be noted that lung burden from the aspiration study was approximately 3 fold greater than with inhalation, i.e., 80 and 28 ug, respectively. Therefore, on an equivalent lung burden basis, inhaled MWCNTs are 3 fold more fibrogenic than MWCNTs delivered by bulk aspiration. Although some may find this surprising, the potency difference may reflect greater dispersion of the dry aerosol vs. MWCNTs in a biologically compatible fluid, resulting in delivery of a greater number of small MWCNT structures per equal mass.

Table 1 provides a comparative breakdown of the distributions of lung burden between aspiration and inhalation of MWCNTs at 1 day post-exposure and their respective fibrillar collagen responses. Although the bolus aspiration dose (80 ug) was nearly 3-fold higher than the lung burden achieved by inhalation exposure (28 ug), only 8 percent of initial lung burden, versus 21 percent for inhalation, was found in the alveolar interstitium. Both exposures had comparable percentage distributions to airway macrophages and the subpleural alveolar tissue. In spite of the 3-fold difference in lung burden between the two exposures, the higher lung burden for aspiration was counterbalanced by higher efficiency of delivery to the alveolar interstitium for inhalation exposure. This is reflective of the greater number of small MWCNT structures in the dry aerosol compared to the liquid aerosol. The net result was to produce similar initial burdens to the alveolar interstitium at one day post-exposure. This effect was not limited to just the 1 day post-exposure as the inhalation exposure lungs at later time points continued to develop higher percentages of the lung burden delivered to the alveolar interstitium. Thus the alveolar interstitial lung burden of inhalation-exposed mice increased even as clearance from the lungs progressed.

**Table 1:** Comparison of Initial Alveolar Interstitial Burden between Bolus aspiration and inhalation of MWCNT

	**Bolus aspiration**	**Inhalation**
Initial lung burden	80 ug	28.1 ug^1^
(ug)		
	64% Alveolar macrophage	58% Alveolar macrophage
Initial distribution	9% Alveolar airspace	6% Alveolar airspace
(%)	**8% Alveolar Interstitium**	**21% Alveolar Interstitium**
	18% Airway	14% Airway
	Macrophage & Airspace	Macrophage & Airspace
	~ 1% Subpleural tissue	~1% Subpleural tissue
Initial Alveolar		
Interstitial burden	6.4 ug	5.9 ug
(ug)		

^1^This inhaled lung burden in the mouse is equal to the predicted human lung burden on an equivalent alveolar surface area basis for a person performing light work at 7 ug/m^3^ for 13 years [3].

The comparable fibrotic responses between bolus aspiration and inhalation of MWCNT in spite of the significant differences in lung burden appear to be in large part to differences in effective delivery of the lung burden to the alveolar interstitium. Although relatively well dispersed on administration, the bolus nature of the aspiration is likely to produce a greater degree of MWCNT agglomeration on the lungs surface as higher concentrations of MWCNTs on the alveolar surface produced by the short duration of exposure are more likely to agglomerate.

## Discussion

Studies of inhaled particle clearance from the lungs are usually based on a rapid phase in which airway clearance occurs, usually within 24 hours after the termination of exposure, and a second slower phase in which particles deposited in the deep alveolar region of the lung are cleared via macrophages [19,20]. Clearance from the alveolar region is slower and occurs as particle-laden macrophages move up the mucociliary escalator or via the lymphatic to the tracheobronchial lymph nodes [21] where the particles not retained by the lymph nodes are released into the venous system at the junction of the jugular and subclavian veins. Insoluble particles, which are not removed by these processes but remain in the airspaces are frequently walled off by foreign body responses mediated by an epithelioid macrophage reaction, forming granulomas [22]. Fibers are also cleared from the intrapleural space via ducts in the parietal pleura which drain into the lymphatics.

However, Donaldson *et al.*[23,24] proposed that long fibers are retained in the stomata which leads to inflammation, fibrosis and potentially mesothelioma. As reviewed by Donaldson *et al*. [25], this pathway may be particular important as CNT seem to have a special ability to stimulate mesenchymal cell growth and to cause granuloma formation and fibrogenesis. Indeed, Takagi *et al.*[26] reported that intraperitoneal injection of MWCNT (3 ug/mouse) led to mesothelioma of the abdominal wall. Murphy *et al.*[24] reported persistent inflammation and fibrosis of the parietal pleural surface 24 weeks after intrapleural injection of long (>15 um) but not short (<4 um) MWCNT (5 ug/mouse). Recently, Schinwald et al. [27] determined the threshold fiber length which would result in pleural inflammation 1 week after intrapleural injection of silver nanofibers of well defined lengths. Results showed a distinct length threshold, with fibers longer than 4 um leading to a pathogenic response. The MWCNT fibers aerosolized in the current study have a mean length of 4.3 um [16]. Given that the mean length of MWCNT fibers in the present study is at the threshold for pathogenic response, it is possible that some effects, such as reduced clearance from the lungs may be due to these mechanisms. As described in the results approximately 1.2 percent of the lung burden was found to translocated to the subpleural tissue and/or visceral pleura in singlet form. This corresponds to 1.2 percent of the 28.1 micrograms of lung burden 1 day post-exposure (see Results) being translocated to the pleural compartment, or approximately 0.35 micrograms, which is an order of magnitude below the burdens at which the aforementioned studies found length-dependent pathologic effects. NIOSH is currently conducting a follow-up exposure to inhaled MWCNT to determine if fiber burden in pleural tissue results in inflammation, lesions and/or transformation of mesothelial cells.

Our studies indicate that the lung burden of MWCNTs is cleared from the alveolar region using these routes. However, the clearance is far from complete at extended times. At 336 days, 65 percent remained in the lungs (Figure 3). This material was most frequently either in smaller MWCNT structures in the alveolar interstitium or larger MWCNT structures associated with macrophages and a smaller but significant component due to MWCNTs in airways at 336 days post-exposure following inhalation (Figure 3). The slow clearance from the airways after inhalation exposure is one of the few differences between our prior aspiration studies [11,28], where airways were largely cleared of MWCNTs by 14 days, and the inhalation studies of this paper. The significantly greater MWCNT retardation in inhalation-exposed lungs is likely due to differences in how the initial MWCNTs in the airspaces of the alveolar region are processed. In aspiration studies there was a much higher initial proportion of large MWCNT structures in the airspaces of alveoli (not directly contained by macrophages). These large airspace MWCNT structures were either cleared up the mucocilliary escalator or enveloped into granulomas by the foreign body response of epithelioid macrophages. In inhalation, the initial percentage of airspace MWCNTs was lower, since the MWCNT structures were much smaller. These smaller MWCNT structures were rapidly incorporated into the alveolar interstitium where clearance is low.

The rapid decline of lung burden for larger MWCNT structures (Figure 5), the relatively unchanged lung burden for singlet MWCNT structures (Figure 5), the increase in percentage of lung burden distributed to the alveolar tissue compartments at 168 days (Figure 3), and the microscopic observations that fibrotic regions were associated with singlets and smaller MWCNT structures (Figure 7) suggests that there is a disassociation of larger MWCNT structures into small or singlet MWCNT structures. In a parallel study of mice from this exposure, we have recently determined that a significant extrapulmonary transport of singlet MWCNTs (~8%) occurs from these lungs [29]. The absence of any significant decline in singlet MWCNTs in the lungs coupled with the demonstration of extrapulmonary transport of singlet MWCNTs indicates that a source of single MWCNTs exist in the lungs. Only disassociation of the MWCNT structures containing multiple fibers into smaller fiber structures has the capacity to simultaneously supply 8% of initial lung burden to the lymph nodes and maintain the burden of singlet MWCNTs in the lungs at slightly increased levels.

A long debated issue in pulmonary toxicology is the relevance of bolus exposure to particles by intratracheal instillation or pharyngeal aspiration to pulmonary responses resulting from inhalation exposure. We have previously reported that at nearly equivalent lung burdens of MWCNTs, pulmonary inflammation after pharyngeal aspiration and whole body inhalation was similar at 1 day post-exposure [3]. The current study supports this conclusion in that the number of PMNs harvested 1 day after a 12 day inhalation to MWCNTs was not significantly different than the PMN response 1 day after pharyngeal aspiration at a similar lung burden [12]. In the present study, we extend this comparison to a later post-exposure time. Specifically, at 28 days post-exposure, comparison of PMNs after pharyngeal aspiration [12] to those in this study shows that at a similar lung burden MWCNT inhalation induced approximately 4-fold greater pulmonary inflammation in comparison to mice exposed by pharyngeal aspiration. This discrepancy is because pulmonary inflammation after pharyngeal aspiration decreased at a greater rate than that following inhalation exposure to MWCNT. The basis for this difference may be related to two factors.

First, although the MWCNTs used in the pharyngeal aspiration study were well dispersed, they still had some larger structures as evidenced by the presence of granulomas in MWCNT-exposed mice [12]. Granulomas were not observed in the lungs of mice exposed by inhalation exposure in the present study as discussed above. MWCNT structures in the inhalation exposed lung were smaller, and thus more particles per mass were present in the lungs. In addition, as a percentage of lung burden, the MWCNT structures were decreasing in size, maintaining a relatively constant number of MWCNT singlets present in the lung (Figure 5). This disassociation process likely contributes to pulmonary inflammation, resulting in a slowly decreasing but more persistent inflammatory response in the lung.

As we described in the results, despite a nearly 3-fold difference in total lung burden between aspiration and inhalation, the alveolar fibrotic response in the post-exposure period was comparable in both its progression over time and magnitude of response. This appears to be due to the comparable disposition of the lung burden to the alveolar interstitial space by the two different routes of exposure. Of note, alveolar airspace granulomas were present in bolus aspiration exposed lungs but were absent in inhalation exposed lungs, yet both bolus aspiration and inhalation exposed lungs have comparable reactions in the alveolar wall. This occurred in spite of the fact that collagen was contained in granulomas. However, granulomatous collagen was specifically excluded from the fibrillar collagen responses given in Figures 7 and 8. The observation that the two routes of administration yield similar fibrotic response in the alveolar wall at similar doses of lung burden to the alveolar wall suggests that the granulomas with encased MWCNT structures do not significantly alter the alveolar interstitial fibrotic response to MWCNTs.

There are a number of possible causes for the bolus aspiration having a much lower final incorporation into the alveolar interstitium. Of these, the bolus or rapid administration and the uniformity of the initial dispersion on the alveolar surface are likely factors. In the bolus administration all of the MWCNTs are distributed on the surface at one time, greatly facilitating nanotube-nanotube interactions. In the inhalation conducted over 12 day less than 10% of the total burden are distributed on the surface on each day of exposure. The greater effective distance between the MWCNT structures during the inhalation exposures may reduce the likelihood of interaction and agglomeration of the MWCNTs before the MWCNTs translocate from the alveolar surface. Although the bolus method was suspended in a dispersion medium and well separated by sonication at the time of administration, the aerosol formation of material produced by the aspiration maneuver [30] essentially separates the MWCNT solution into a respirable fluid aerosol which contains larger MWCNT structures than the inhalation exposure where the CMAD was 420 nm. These larger MWCNT structures deposited by aspiration are not as rapidly incorporated into the alveolar interstitium as the smaller MWCNT structures.

Results coupling the measurement of MWCNT lung burden with the distribution of MWCNTs in different lung regions (Figure 3) and the size dependence of clearance of MWCNT structures (Figure 5) reveals that clearance from the lungs is neither a simple one or two component process. Lung clearance exhibits multiple distinct and interacting components, i.e., rapid clearance from the airways to a low steady-state level, slow clearance from the alveolar region of the lung by the decline in burden of alveolar macrophages, and an increase in the MWCNT burden incorporated into alveolar tissue (Figure 3). As shown in Figure 5, there were compositional changes of MWCNTs within these compartments as well. The lung burden of singlet MWCNTs and smaller MWCNT structures, which are predominately within the alveolar interstitial space, increased over the 168 day post-exposure period examined (Figure 5). There was also significant, but low level content of MWCNTs remaining in the airways due to the presence of macrophages loaded with MWCNTs. The results clearly demonstrate that the redistribution and clearance of MWCNTs from the lungs is not simply the decline of all MWCNT fibers from a single uniform compartment.

The inhalation exposure in the present study resulted in a lung burden of 28 ug/lung in mice. This resulted in a substantial inflammatory and injury response (Figure 6), which decreased with time post-exposure, and a progressive fibrotic response (Figure 8). Recently our laboratory conducted an inhalation study using the same MWCNT sample which resulted in lung burdens of 0.31, 3.1 and 31.0 ug/mouse [31]. As expected, dose-dependent pulmonary responses were seen. At the lowest lung burden, pulmonary responses were minimal with only small changes in induction of selected inflammatory genes. A full manuscript describing these results is in preparation.

## Conclusions

Inhalation exposure to MWCNT produced a fibrotic response that was found to develop and persist out to 336 days after exposure, which is significantly longer that examined in prior bolus aspiration studies. This study also found large differences in the clearance rates from the lungs depending on the size of the MWCNT structures. Clearance of larger MWCNT structures containing greater than 4 fibers from the lung predominately occurs by reduction in the lung burden of MWCNTs within alveolar macrophages. Smaller MWCNT structures in general and singlet MWCNT in particular are formed from dissociation of larger structures and are retained principally within the alveolar septa and sub-pleural tissue. Singlet MWCNTs and smaller MWCNT structures retained within the alveolar septa produce a progressive fibrotic reaction in the lungs.

## Methods

### Animal

Male C57BL/6 J mice (7 weeks old) were obtained from Jackson Laboratories (Bar Harbor, ME). Mice were housed one per cage in polycarbonate ventilated cages, which were provided HEPA-filtered air, with fluorescent lighting from 0700 to 1900 hours. Autoclaved Alpha-Dri virgin cellulose chips and hardwood Beta-chips were used as bedding. Mice were monitored to be free of endogenous viral pathogens, parasites, mycoplasms, Helicobacter and CAR Bacillus. Mice were maintained on Harlan Teklad Rodent Diet 7913 (Indianapolis, IN), and tap water was provided ad libitum. Animals were allowed to acclimate for at least 5 days before use. All animals used in this study were housed in an AAALAC-accredited; specific pathogen-free, environmentally controlled facility. All animal procedures were studied under Protocol # 10-DP-M-008 approved by the NIOSH Institutional Animal Care and Use Committee.

### Carbon nanotube source

MWCNTs used in this study were obtained from Hodogaya Chemical Company (MCWNT-7, lot #061220-31) and were manufactured using a floating reactant catalytic chemical vapour depositon method followed by high thermal treatment in argon at 2500°C furnace. This lot of MWCNT-7 was fully characterized in our prior report in which acute inhalation exposures were conducted with this lot of MWCNTs [3]. Briefly, MWCNT trace metal contamination was 1.32%, with iron (1.06%) being the major metal contaminants. Average MWCNT surface area measured by nitrogen absorption-desorption technique (Brunauer-Emmett-Teller method, BET) was 26 m^2^/g [11].

### MWCNT aerosol generation and aerosol characterization

Mice were exposed to a MWCNT aerosol (5 mg/m^3^, 5 hours/day) for 12 days, using an acoustical-based computer controlled system designed and constructed by our laboratory [10] Details of the exposure system, aerosol control performance and aerosol characterization have previously been published in detail [3]. In brief, the inhalation exposure system combines air flow controllers, aerosol particle monitors, data acquisition devices, and custom software with automated feedback control to achieve constant and repeatable exposure chamber temperature, relative humidity, pressure, aerosol concentration, and particle size distributions. The generator produces airborne particles continuously for long periods of time, e.g. 35 hours of continuous operation, with minimal fluctuations during an exposure period. The uniformity of test atmosphere in the chamber was evaluated to have a total variation of < 5%. In this study, the MWCNT aerosol mass concentration was continuously monitored with a Data RAM (DR-40000 Thermo Electron Co, Franklin, MA), and gravimetric determinations (37 mm cassettes with 0.45 μm pore-size Teflon filters) were used to calibrate and verify the Data RAM readings. The mass mode aerodynamic diameter was 1.3 μm with a count mode aerodynamic diameter of 0.42 μm [3]. When characterized by lognormal statistics, the distribution was shown to have a mass median aerodynamic diameter (MMAD) of 1.5 μm and a geometric standard deviations (GSD) of 1.67 [3]. As described previously [3], the resultant inhaled lung burden in the mouse from this exposure is equal to the predicted human lung burden on an equivalent alveolar surface area basis for a person performing light work at 7 ug/m^3^ for 13 years.

#### Lung fixation and section preparation

At 1 day, 14 days, 84 days, 168 days and 336 days after the 12 day exposure period, mice were anesthetized, euthanized by an overdose of pentobarbital (>100 mg/kg body weight, i.p.) and lungs and systemic tissues were preserved by whole body vascular perfusion of paraformaldehyde while the lungs were inflated with air via a tracheal cannula. Separate, clean-air control groups were studied. Following fixation the diaphragm, heart, kidney, liver and brain were removed and sliced into 2–3 mm thick tissue blocks. The left lung and sliced tissue blocks were embedded in paraffin. The chest wall was decalcified in formic acid prior to embedding. Sections (5 micron thick) were collected on ultrasonically cleaned, laser cut slides (Schott North America, Inc, Elmsford, N.Y. 10523) to avoid nanoparticle contamination from the ground edges of traditional slides. To enhance the contrast between tissue and MWCNTs, sections were stained with Sirius Red. Sirius Red staining consisted of immersion of the slides in 0.1% Picrosirius solution (100 mg of Sirius Red F3BA in 100 ml of saturated aqueous picric acid, pH 2) for 1 hour followed by washing for 1 minute in 0.01 N HCl. Sections were then briefly counterstained in freshly filtered Mayer’s hematoxylin for 2 minutes, dehydrated, and coverslipped. For serial section analysis of lung clearance of MWCNT fibers and the number of MWCNT fibers per MWCNT structure in the lungs, three serial sections of the lungs were collect on one slide and stained as described above.

#### MWCNT lung burden

At 1, 14, 28, 84, 168 and 336 days post-exposure, mice were euthanized with an i.p. injection of sodium pentobarbital (>100 mg/kg body weight) followed by exsanguination. After euthanasia, lungs were removed and stored at −80°C. MWCNT burden determinations were conducted using a method previously described by our laboratory [3,32] with minor modification. The lung tissue was digested in 25% KOH/methanol (w/v) at 60°C overnight, followed by centrifugation at 16,000 × g for 10 minutes. The supernatant was removed, the remaining pellet was mixed with 50% HNO_3_/methanol (v/v), and incubated at 60°C overnight, followed by centrifugation (16,000 × g, 10 minutes). The supernatant was removed, and the pellet resuspended in 10% NP-40 (v/v) in dH_2_O, followed by 60 second sonication (≈3,100 Joules) using water-cooled cup horn sonicator. MWCNT standards were processed in parallel with the lung samples. The optical densities of the solutions were measured at 700 nm using a UV/visible spectrophotometer. Lung MWCNT content was determined from a standard curve.

#### Whole lung lavage

At 1, 14, 28, 84, 168 and 336 days post-exposure, mice were euthanized with an i.p. injection of sodium pentobarbital (>100 mg/kg body weight) followed by exsanguination. A tracheal cannula was inserted and bronchoalveolar lavage (BAL) was performed through the cannula using ice cold Ca^2+^ and Mg^2+^-free phosphate buffered saline, pH 7.4, supplemented with 5.5 mM D-glucose (PBS). The first lavage (0.6 ml) was kept separate from the rest of the lavage fluid. Subsequent lavages, each with 1 ml of PBS, were performed until a total of 4 ml of lavage fluid was collected. BAL cells were isolated by centrifugation (650 × g, 5 minutes, 4°C). An aliquot of the acellular supernatant from the first BAL (BAL fluid) was decanted and transferred to tubes for analysis of lactate dehydrogenase (LDH) and albumin. The acellular supernatants from the remaining lavage samples were decanted and discarded. BAL cells isolated from the first and subsequent lavages for the same mouse were pooled after resuspension in PBS, centrifuged a second time (650 × g, 5 min, 4°C), and the supernatant decanted and discarded. The BAL cell pellet was then resuspended in PBS and placed on ice. Total BAL cell counts were obtained using a Coulter Multisizer 3 (Coulter Electronics, Hialeah, FL) and cytospin preparations of the BAL cells were made using a cytocentrifuge (Shandon Elliot Cytocentrifuge, London). The cytospin preparations were stained with modified Wright-Giemsa stain, and cell differentials were determined by light microscopy.

#### BAL fluid LDH activity and albumin

BAL fluid LDH activities were evaluated as a marker of cytotoxicity. BAL fluid LDH activities were determined by monitoring the LDH catalyzed oxidation of lactate to pyruvate coupled with the reduction of NAD^+^ at 340 nm using a commercial assay kit (Roche Diagnostics Systems, Montclair, NJ). Both the BAL fluid albumin and LDH assays were conducted using a COBAS MIRA Plus (Roche Diagnostic Systems, Montclair, NJ).

#### Field emission scanning electron microscopy

For scanning electron microscopy, sections of the lung were cut at 8 microns, placed on carbon planchets, deparaffinized and sputter coated. After coating, the specimens were examined with a Hitachi Model S-4800 Field Emission Scanning Electron Microscope (FESEM) at 5 to 10 kV and working distances of 4.5 mm to 6 mm for magnifications of 100,000× to 1000×, respectively. Photographs were taken in slow scanning mode at 1280 × 1024 pixels. Use of thin sections from paraffin embedded tissue was found to be preferable to large, unevenly cut blocks because it provided a uniform thickness of organic material on the carbon planchet. The 8 micron sections were thick enough to convey three-dimensional information but still thin enough to avoid effects due to charging and/or undergoing physical shifts when examined at the high magnifications necessary to study nanomaterials.

#### Enhanced-darkfield light microscopy imaging of MWCNTs

Carbon nanotubes in sections from exposed lungs were assessed using an enhanced-darkfield optical system. Nanomaterials, such as carbon nanotubes, have dimensions less than the wavelength of light, have closely packed atoms, and typically have a refractive index significantly different from that of biologic tissues and/or mounting medium. These characteristics produce significantly greater scattering of light by nanoparticles than by the surrounding tissues. The enhanced-darkfield optical system images light scattered in the section and, thus, nanomaterials in the section stand-out from the surrounding tissues with high contrast. Using this method of imaging, it is practical to scan whole lung sections at relatively low magnification (40-60× objectives) to identify CNTs that would not be detected by other means.

The optical system consisted of high signal-to-noise, darkfield-based illumination optics adapted to an Olympus BX-41 microscope (CytoViva, Auburn, AL 36830). Sections for dark-field examination were specifically cut from paraffin blocks and collected on ultrasonically cleaned, laser cut slides (Schott North America Inc, Elmsford, N.Y. 10523) to avoid nanoparticle contamination from the ground edges of traditional slides. After staining with Sirius Red-Hematoxylin, sections were coverslipped with Permount. After alignment of the substage oil immersion optics with a 10× objective, sections were examined with 60× air or 100× oil immersion objectives. Enhanced darkfield images were taken at 2400×4800 pixels with a Olympus DP-73 digital camera (Olympus America Inc., Center Valley, PA 18034).

#### Lung distribution of MWCNTs

The distribution of MWCNTs in the lungs was determined by counting the occurrence of MWCNTs under an eyepiece point counting overlay using standard morphometric point counting methods [33] as previously described for study of the distribution of CNTs [11,34]. Point counting categories were subdivided into points over MWCNTs in airway region, points over MWCNTs in alveolar regions, and points over MWCNTs in the subpleural tissue region. Airway regions were defined as those containing airway tissue (airway epithelial cells-basement membrane and tissues of the broncho-vascular cuff), airway lumen, and associated blood vessels greater than 25 microns. Alveolar regions were those containing alveolar tissue and alveolar air space. The subpleura tissue region included MWCNTs in the subpleural tissue and MWCNTs in the visceral pleural surface. The subpleural tissue regions included the immediately subpleural alveolar interstitial-epithelium layer and subpleural lymphatics but did not include any portion of alveolar walls attaching to the pleura. Points in airway and alveolar regions were further subdivided into points over MWCNTs that were in the airspace, points over MWCNTs that were in tissue of the region, and points over MWCNTs that were partially or completely within macrophages.

To accomplish the counting, an eyepiece counting overlay consisting of 11 by 11 lines (121 total points for each throw of the overlay) was used with a 100× oil immersion objective. A grid pattern for throws of the counting overlay was used in order to ensure a uniform sampling of the section which did not overweight interior points. The counting overlay throws of the eyepiece were positioned over the section at 12 uniformly spaced grid points in both X and Y co-ordinates. These 12 grid points were determined using the stage micrometer scale to measure the X and Y bounds of the section. Using the bounding rectangle of these co-ordinates, a 3 by 4 grid was selected and the 12 intersections were used as the center point for each of the eyepiece counting overlay throws.

For each animal, three sections were counted, and the counts for the airways, alveolar and subpleural tissue regions were summed. Each counting category was divided by this total and multiplied by 100 to express the results as a percentage of total lung burden. To express the results in terms of absolute lung burden (ug), each percentage was then multiplied by 28 ug/lung based on measurements of initial total lung burden of MWCNT in the results Eight animals were analyzed per group. Clean-air controls were also cut and scanned for MWCNTs but none were found. Additional, negative controls to test for contamination by MWCNTs from the treated lungs included changes of the embedding processor solutions between treatment groups, changes of sectioning bath between blocks and use of a new sectioning knife edge for each block.

#### MWCNT fiber number per MWCNT structure

Serial section analysis at 1 day, 14 days and 168 days was used to measure the clearance of MWCNT fibers from the lungs and to assess how the composition of MWCNT structures was altered by redistribution and clearance. For each series of sections a series of 6 overlapping photographs were taken at 100× (oil immersion) using the enhanced darkfield optical system. Each MWCNT structure was identified in prints of the middle section. The prints from all three sections of the series were then used to determine if the MWCNTs identified in the middle section contained one, two, three, four or more than four fibers. Point counting of the middle section was then used to determine the volume density of MWCNTs for each fiber class (one, two, three, four and more than four fibers), total volume density of all MWCNT fibers, and the relative distribution in terms of fibers per MWCNT structure.

#### Morphometric analysis of collagen distribution

Morphometric analysis was conducted to determine the fibrillar collagen response to inhalation of MWCNTs at 1, 14, 84, 168 and 336 post-exposure and in clean-air controls. Collagen fibers in the lungs were detected with Sirius Red staining [35], which has been demonstrated to be a quantitative morphometric method for collagen fiber determination in the lungs [36,37]. Quantitative morphometric methods were used to measure the average thickness of Sirius Red positive connective tissue fibers in the alveolar regions. Volume and surface density were measured using standard morphometric analyses [38,39]. This consisted of basic point and intercept counting. Volume density was determined from counting the number of points over all tissues in the alveolar regions and points over Sirius Red positive connective tissue. Surface density of the alveolar wall was determined from intercepts between a line overlay and the alveolar wall. These point and intercept counts were made using a 121-point /11-line overlay graticule (12.5 mm square with 100 divisions), at 100× magnification, taken at six locations equally spaced across each section (one section per animal). This process was repeated twice for each animal. In order to limit the measurements to alveolar parenchyma, areas containing airways or blood vessels greater than 25 mm in diameter were excluded from the analysis. Average thickness of the Sirius Red positive connective tissue fibers of the alveolar wall was computed from two times the ratio of volume density of point to the surface density of the alveolar wall. Mean linear intercept length, a measure of the average size of the alveolar/alveolar duct airspaces dimensions in the alveolar region, was computed from the ratio of volume density to surface density [38].

### Statistical analyses

Data were analyzed using analysis of variance (STATGRAF). Bartlett’s test was used to test for homogeneity of variances between groups. Statistical differences were determined using one-way analysis of variance with significance set at p ≤ 0.05. When significant F values were obtained, individual means were compared to clean-air controls using Duncan’s multiple range test [40], and P < 0.05 was considered to be significant. Data are given as Means ± S.E.

For the BAL data statistical comparisons between MWCNT and air controls were performed separately for each post-exposure time using analysis of variance (ANOVA). Since variance estimates were different across treatment groups, the ANOVA models were estimated using an unequal variance method available from SAS PROC MIXED [41]. Similarly, comparisons across post-exposure times were performed using ANOVA and post-hoc comparisons using the Tukey method to account for multiple comparison. All statistical tests were two tailed with significance level equal to 0.05. All statistical tests were two-tailed and performed at the 0.05 significance level.

## Abbreviations

BET: Brunauer-Emmett-Teller method, BMD, Benchmark dose; CNT: Carbon nanotube; DM: Dispersion medium; DPPC: 1,2 dipalmitoyl-sn-glycero-3-phosphocholine; FESEM: Field emission scanning electron microscope; GSD: Geometric standard deviations; MMAD: Mass median aerodynamic diameter; MWCNTs: Multi-walled carbon nanotubes; PBS: Phosphate-buffered saline; TEM: Transmission electron microscope.

## Competing interests

The authors declare that they have no competing interests.

## Authors’ contributions

RM conceived of the study, developed the morphometric methods, conducted the FESEM evaluation, analyzed the experimental results and drafted the manuscript. JS performed the morphometric counting and assisted in analysis of results. AH was involved in the planning and writing of the manuscript. LB provided important information on sampling of the lungs for study and conducted lung preparation for histopathology. WM designed and developed the MWCNT aerosol exposure system. SF assisted in the sampling design and operation of the CytoViva studies. MW conducted whole lung lavage studies. MA developed the experimental design. VC and DP contributed to the experimental design, acquisition of funding and writing of the manuscript. All authors read and approved the final manuscript.
